# In-Hospital Outcomes Following Surgical Revascularization of Chronic Total Coronary Occlusions

**DOI:** 10.3390/medicina59111967

**Published:** 2023-11-08

**Authors:** Albi Fagu, Tim Berger, Clarence Pingpoh, Stoyan Kondov, Maximilian Kreibich, Jan Minners, Martin Czerny, Matthias Siepe

**Affiliations:** 1 Department of Cardiovascular Surgery, Heart Centre Freiburg-Bad Krozingen, Faculty of Medicine, University of Freiburg, 79085 Freiburg, Germanymaximilian.kreibich@uniklinik-freiburg.de (M.K.);; 2 Division of Cardiac Surgery, University Hospital “Shefqet Ndroqi”, University of Medicine, 1005 Tirana, Albania; 3 Department of Cardiac Surgery, University Hospital Bern, University of Bern, 3012 Bern, Switzerland; 4 Department of Cardiology and Angiology II, University Heart Centre Freiburg-Bad Krozingen, 79189 Bad Krozingen, Germany

**Keywords:** chronic total occlusion, coronary revascularization, CABG

## Abstract

*Background and Objectives*: Patients with chronic total occlusions of the coronary arteries are either treated with PCI or referred for surgical revascularization. We analyzed the patients with chronic occluded coronary arteries that were surgically treated and aimed to describe the anatomical characteristics, revascularization rates, and in-hospital outcomes achieved with coronary artery bypass grafting. *Methods*: Angiographic data of 2005 patients with coronary artery disease treated in our institution between January 2005 and December 2014 were retrospectively analyzed. A total of 1111 patients with at least one coronary total occlusion were identified. We reviewed the preoperative coronary angiograms and surgical protocols to determine the presence, localization, and revascularization of coronary occlusions. We also evaluated the perioperative data and in-hospital outcomes. *Results*: The median age of the study population was 68 years (25th–75th percentiles, 61.0–74.0). Three-vessel disease was present in 94.8% of patients and the rest (5.8%) had a two-vessel disease. The localizations of the occlusions were as follows: 68.4% in the RCA system, 26.4% in the LAD, and 28.5% in the LCX system. Multiple occlusions were present in 22.6% of the patients. Complete coronary total occlusion revascularization was achieved in 86.1% of the patients. The overall in-hospital mortality was 2.3%. The median in-hospital stay was 14.0 days. After logistic regression analysis, age (odds ratio 3.44 [95% confidence interval, 1.81–6.53], *p* < 0.001, for a 10-year increase) and the presence of peripheral artery disease (odds ratio 3.32 [1.39–7.93], *p* = 0.007) were the only statistically significant independent predictors of in-hospital mortality. *Conclusions*: A high revascularization rate and favorable in-hospital outcomes are achieved with coronary artery bypass surgery in patients with multi-vessel diseases and coronary total occlusions. Older age and the presence of peripheral artery disease are independent predictors of in-hospital mortality. A long-term follow-up and the type of graft (arterial vs. venous) used would bring more useful data for this type of revascularization.

## 1. Introduction

Chronic total occlusions (CTOs) are a common finding in patients with coronary artery disease (CAD) [1,2,3]. It has a predilection for the right coronary artery (RCA) and almost equally affects the left anterior descending (LAD) and left circumflex (LCX) arteries. The occlusions are mostly located in the proximal or middle segment of the artery, but also in the distal portion in the case of the LCX artery [4]. In the presence of collateral circulation, the occluded vessel is mainly perfused by either the ipsilateral or the contralateral coronary artery. This helps in preventing myocardial infarction and in preserving the left ventricular function in as much as 50% of cases [1,5].

Three treatment options are considered for these patients: coronary artery bypass grafting (CABG), percutaneous coronary intervention (PCI), or optimal medical treatment [6,7,8]. Based on the current literature, patients with multi-vessel diseases (often in the presence of CTOs) are better treated with surgery [8,9,10]. In reality, only 25–40% of these patients are referred for surgery and the rest receive CTO-PCI or medical treatment [11]. Furthermore, for the CTO-PCI indications, currently, only symptoms refractory to treatment support the indication for revascularization [8,11,12].

The CTO prevalence in the surgical population considerably varies in the medical literature as it is subject to geographical differences in treatment strategies [6,7]. While the literature on the treatment of CTOs with PCI is extensive, the evidence regarding the outcomes of surgical revascularization in patients with CTOs is limited.

Our objective was to analyze the clinical outcomes of patients with CTOs referred to surgery and to describe the anatomical characteristics and revascularization rates achieved with CABG.

## 2. Materials and Methods

### 2.1. Patients

Between January 2005 and December 2014, 2005, consecutive patients treated with CABG for CAD at the University Heart Center Freiburg were retrospectively evaluated. Patients with previous cardiac operations, concomitant non-CABG surgery, and acute coronary syndrome were excluded. A total of 1111 patients (62.7%) with at least one CTO were included in this analysis.

### 2.2. Morphological Coronary Analysis

Preoperative invasive coronary angiograms (ICAs) were reviewed to determine the presence and localization of coronary occlusions. The AHA committee reporting system was used to describe the localizations of CTOs [13]. Small, occluded vessels (≤1.5 mm) were considered not revascularizable, and these patients were excluded from the analysis [14].

### 2.3. Data Collection

Patient characteristics, disease-specific variables, comorbidities, surgical variables, and laboratory data were extracted from the patient medical records. The surgical protocols were checked for intraoperative variables and to find if the CTOs were bypassed. Full CTO revascularization was defined as the successful revascularization of every CTO.

The primary study endpoint was in-hospital all-cause mortality. The secondary endpoint was the successful revascularization of CTOs.

### 2.4. Definitions

Significant CAD was defined as ≥1 coronary artery with a ≥70% stenosis. A total occlusion (interrupted antegrade flow) was considered chronic when it was ≥3 months old [15]. The definition of in-hospital mortality was death occurring during the same hospitalization. For the definition of a perioperative myocardial infarction, we used the 4th universal definition for procedure-related myocardial infarctions (Type 5 MI).

### 2.5. Statistical Analysis

Descriptive statistics for continuous variables are presented as median (interquartile range). The number of anastomoses per patient is presented as mean ± standard deviation for better comparison with other previously published studies after normality was graphically verified using Q-Q plots. Categorical data are presented as absolute and relative frequencies. Exploratory hypothesis testing of group differences among demographics, clinical, lesional, and surgical characteristics, as well as postoperative variables, were performed with non-parametric Wilcoxon signed-rank tests for continuous data and χ^2^ tests or Fisher’s exact tests in cases of an expected cell size of <5 for categorical data. A logistic regression model was used to identify the independent correlates of in-hospital mortality. Covariates entered into the multivariable models were selected using LASSO (Least Absolute Shrinkage and Selection Operator). The selected variables included age, gender, the presence of chronic obstructive pulmonary disease or peripheral artery disease, reduced left ventricular ejection fraction, and the use of the bilateral internal mammary artery. Risk estimates are presented as odds ratios (ORs) with 95% confidence intervals (CIs). Two-sided *p* values < 0.05 were considered to show statistical significance. The statistical analyses were performed using R 3.6 Statistical Software (R Foundation for Statistical Computing).

## 3. Results

### 3.1. Baseline Characteristics

The baseline characteristics are shown in Table 1. The median age was 68 years [61.0–74.0]. The non-survivors were significantly older: 76 years [70.0–79.0] vs. 68.0 years [61.0–73.8] (*p* < 0.001). Most patients were males (85.2%) with no difference between both groups (*p* > 0.99). The presence of hypertension, diabetes, smoking history, and hypercholesterolemia was comparable in both groups. Preoperative atrial fibrillation was present in 8.2% (8.0% vs. 16%, *p* = 0.142) and 29.7% of them had a prior myocardial infarction (29.6% vs. 36.0%, *p* = 0.634). COPD and prior stroke were comparable in both groups. Peripheral artery disease (PAD) was present in 14% of the patients (13.4% vs. 36.0%, *p* = 0.004) with a statistically significant difference. The majority (94.8%) of our patients had three-vessel disease with no difference between the groups (*p* > 0.99). The left ventricular function was severely reduced (LVEF < 35%) in 4.4% of our study population, but the numerical difference between survivors and non-survivors (4.2% vs. 12.0%, *p* = 0.094) did not reach statistical significance. The median EuroSCORE was 1.43 [1.02;2.33] with a significant difference between the two groups [1.42 (1.02;2.30) vs. 3.33 (1.56;4.54), *p* = 0.001].

### 3.2. Morphological Coronary Analyses and Revascularization Rates

**Figure 1:**
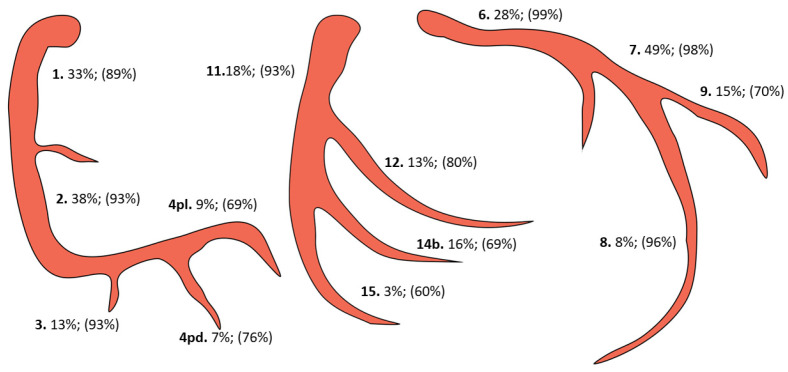
CTO localization and respective revascularization rates (pd, posterior descending; pl, posterolateral).

Morphological coronary characteristics and revascularization rates are reported in Table 2. The detailed description using the AHA Committee reporting system is depicted in Figure 1. The 1111 patients had a total of 1371 CTOs with 1.24 (±0.45) CTO/patient. A total of 1204 of the CTOs (1.08 ± 0.54 CTOs/patient, or 87.8%) were successfully bypassed. There was no significant difference between survivors and non-survivors regarding the number of CTOs or the revascularization rate (*p* = 0.989, *p* = 0.974). In 68.4% of the study population, there was a CTO localized in the RCA system, 26.4% in the LAD, and 28.5% in the LCX system. Multiple CTOs were present in 22.6% of the patients with 8.5% involving the RCA and LAD systems, 10.4% the RCA and LCX systems, 2.7% the LAD and LCX systems, and 1.1% involving all three main coronaries. There was no significant difference between the two groups regarding the CTO localization (RCA *p* = 0.543, LAD *p* = 0.616, LCX *p* = 0.777). RCA-CTOs were revascularized in 89.1% of cases and in 91.2% of cases when the occlusion was located in the first three segments of the RCA system (excluding seg. 4). In the case of LAD-CTOs, the lesions were bypassed in 93.2% of the cases and in 97% of the cases when the CTO was located in the main vessel (excluding segs. 9 and 10). The revascularization rate of the LCX-CTOs was 83.6% for the LCX system and 86.3% when the distal part (segments 14b, 15) was excluded (Figure 1). The revascularization rates of the CTOs in the LCX position were significantly lower when compared with the LAD position (*p* < 0.001) but not when compared with the RCA position (*p* = 0.07). Full CTO revascularization was achieved in 86.1% of patients with no significant difference between survivors and non-survivors (*p* > 0.99).

### 3.3. Surgical Data

Surgical data are presented in Table 3. The median CPB time was 88.0 min [75.0–104.0] with no significant difference between survivors and non-survivors (*p* = 0.276). The median cross-clamp time was 64.0 min [54.0–76.0] for the survivor group and 62.0 [55.0–99.0] for the non-survivors (*p* = 0.695). The number of anastomoses per patient was 3.73 (0.9) (*p* = 0.758). Significantly more patients were treated using a multi-arterial approach (BIMA) in the survivor group: 46% vs. 16% (*p* = 0.005).

### 3.4. Postoperative Data and Outcomes

The overall in-hospital mortality was 2.3% (25 of 1086) and the 30-day mortality was 2% (22 of 1086). The rate of postoperative stroke was 1.71% (*p* = 0.066) and that of postoperative myocardial infarction was 4.41% (*p* = 0.004). New-onset atrial fibrillation occurred in 19.4% of the patients (*p* = 0.449). The length of hospital stay was 14.0 days [11.0–18.0] and was significantly longer in the non-survivor group (*p* < 0.001). No difference was noted regarding surgical re-exploration for bleeding, but non-survivors received more transfusions. More patients in the non-survivor group required postoperative dialysis with a statistically significant difference (*p* = 0.016). The outcome data are summarized in Table 3.

Based on the results of the logistic regression analysis, age (odds ratio 3.44 [95% confidence interval, 1.81–6.53], *p* < 0.001, for a 10-year increase) and presence of peripheral artery disease (odds ratio 3.32 [1.39–7.93], *p* = 0.007) were the only statistically significant independent predictors of in-hospital mortality (Table 4).

**Table 3:** Surgical data and postoperative outcomes.

	All (N = 1111)	Survivors (N = 1086)	Non-Survivors (N = 25)	*p*-Value
CPB Time (min)	88 [75–104]	88 [75–104]	90 [73–128]	0.276
Cross-clamp Time (min)	64 [54–76]	64 [54–76]	62 [55–99]	0.695
Operative Time (min)	210 [190–240]	210 [190–240]	210 [180–250]	0.958
ICU Stay (days)	0.96 [0.87;1.92]	0.96 [0.87;1.91]	5.77 [1.26;14.5]	<0.001
Postop. Stroke	19 (1.71%)	17 (1.57%)	2 (8.00%)	0.066
Postop. MI	49 (4.41%)	44 (4.05%)	5 (20.0%)	0.004
Postop. AFib	215 (19.4%)	212 (19.5%)	3 (12.0%)	0.449
Postop. Bleeding	39 (3.51%)	39 (3.59%)	0 (0.00%)	>0.99
Blood Transfusion (units)	2 [0;4]	2 [0;4]	10 [3;14]	<0.001
Plasma Transfusion	0 [0;3]	0 [0;3]	3 [0;4]	0.002
Postop. Dialysis	31 (2.79%)	13 (1.83%)	18 (4.51%)	0.016
Postop. Troponin ((maximal) g/mL)	0.61 [0.39–1.05]	0.61 [0.38–1.04]	1.54 [0.80–2.04]	<0.001
Postop. Leukocytes (1/μL)	12,895 [10,540;15,880]	12,830 [10,530;15,728]	20,435 [13,842;30,328]	<0.001
Postop. CRP (mg/dL)	23.1 [18.1–27.8]	23.1 [18.2–27.7]	25.5 [11.8–29.1]	0.851
Postop. Creatinine (mg/dL)	1.3 [1.1–1.7]	1.3 [1.1–1.7]	1.5 [1.2–1.7]	0.587
Hospital Stay (days)	14 [11–18]	14 [11–18]	15 [6–23]	0.959
Postop. GOT (U/L)	61 [46;95]	61 [46;91]	206 [113;1063]	<0.001
Postop. GPT (U/L)	49 [33;78]	49 [32;77]	236 [53.2;789]	<0.001
In-hospital Mortality	2.3%			
Thirty-day Mortality	2.0%			

Table 3. Data are presented as median (q1, q3) for continuous variables and as n (%) for categorical variables unless specified otherwise. CPB, cardiopulmonary bypass; ICU, intensive care unit; MI, myocardial infarction; AF, atrial fibrillation; GOT, Glutamate-Oxalacetat-Transaminase; GPT, Glutamate-Pyruvate-Transaminase.

## 4. Discussion

The three most essential findings of this study can be summarized as follows:

- CABG achieves high CTO revascularization rates.
- The surgical treatment of patients with CTOs offers very favorable early outcomes.
- Older age and PAD are predictors for in-hospital mortality.

The treatment of complex CAD has always been a matter of debate between cardiologists and cardiac surgeons. According to the current guidelines, patients with a higher Syntax Score, especially in the presence of a CTO, are better treated with surgery [8]. In reality, only 25–40% of these patients are referred to surgery and the rest receive PCI or medical treatment [16]. The treatment of these patients with CABG does offer high CTO revascularization rates and excellent results. Previous reports on the results of the surgical treatment refer to cohorts with relatively limited numbers (<600) of patients with CTOs [7,14,17]. Regardless of the approach, there is a signal that the revascularization of a CTO can lead to fewer major cardiovascular events during long-term follow-up [18].

Our cohort had similar disease profiles and demographic data compared with those of other studies [1,7]. As expected, our patients had a high prevalence of hypertension, diabetes, hypercholesterinemia, and other cardiovascular risk factors. All patients had a multi-vessel disease with 94.8% of them having a three-vessel disease. Of note, the amount of three-vessel disease in this cohort was higher compared with that of other published studies [7]. This difference may have originated from the high level of expertise in CTO-PCI in our center and an active selection of the cases referred for surgery.

Only a minority of the patients had a severely reduced LV function. As mentioned above, the collateral system plays a protective role in avoiding muscle cell necrosis [1,5]. In other published studies, the LV function was severely impaired in 17–20% of patients [1,6]. We found that only 4.4% had a severely impaired LV function and found no significant difference between the two groups. The tendency to treat patients with severely reduced LV function with PCI in order to reduce adverse outcomes may have influenced these results. The presence of a well-functioning collateral system can prevent myocardial infarction in a considerable number of patients but is not a guarantee for preserved viability. In fact, the principal factors that influence the ensuing myocardial infarction are the rate of progression, duration, adequate collateral flow, and myocardial response to ischemia [5]. Data showing survival improvement after viability-guided revascularization in patients with LV dysfunction have already been published, but other randomized trials have failed to prove its importance [19,20]. We have not routinely tested the viability of the area related to CTOs as we think that it does not influence the treatment of patients with a multi-vessel disease with an otherwise clear indication of surgical revascularization.

At least one CTO was present in 62.7% of all patients treated for stable CAD at our institution. This prevalence was highly attributable to the different treatment strategies chosen in the respective centers and substantially differed between the PCI and CABG cohorts [1,7,16,21]. In this latter cohort, 87.8% of all CTOs were successfully bypassed. CTOs localized in the RCA, LAD, and LCX territories were successfully revascularized in 89.1%, 93.2%, and 83.6% of cases, respectively. Of note, there was a significant difference in the revascularization rate of the LCX-CTOs when compared with the LAD-CTOs. The lower revascularization rates for the LCX artery could be explained by the fact that the occlusions were localized in the distal portion of the vessel in a considerable number of cases (Figure 1) and the surgical revascularization can sometimes be difficult to accomplish [4]. In our experience, the localization of the CTO in the sulcus segment of the LCX artery can pose technical difficulties for the surgeon during revascularization. Full CTO revascularization was achieved in the large majority of patients (86.1%), which prevented a reliable assessment of the impact of non-revascularized CTOs on outcomes. Our results regarding the revascularization rates of CTOs were comparable with those of other surgical registries [1,7]. The reasons for the non-successful revascularization of CTOs in our study included: the small caliber of the occluded vessel, heavily calcified vessel, and surgeon’s preference.

When we excluded the localizations of the CTOs in the distal segments of the RCA, LCX, and LAD systems, or the small branches, the revascularization rates increased considerably. In many previous published reports on the revascularization of CTOs with PCI, the more distal lesions were considered to be not amenable to PCI. In the DECISION-CTO, Euro-CTO, and EXPERT-CTO trials, different inclusion criteria like: “target coronary diameter ≥ 2.5 mm”, or “CTO localized in segments 1–3 for RCA, 6–7 for LAD, 11–12 for LCX” were used to exclude the more distal CTO localizations [22,23,24]. As we have shown above, this can produce severely biased better results in a selected group of patients, and the CTO-PCI probably achieved “high” revascularization rates by avoiding the small, more distal segments.

The use of multiple arterial grafts has been shown to offer survival benefits and is strongly supported by the current guidelines [8]. Nevertheless, total- or multi-arterial revascularization rates worldwide remain considerably low, with 15–30% of CABG patients receiving multiple arterial grafts. Technical difficulties, the increased risk of wound infections, the longer operation time, and the fear of competitive flow are some of the limitations to a wider acceptance of this technique [25,26,27,28]. Multi-arterial revascularization using the right internal mammary artery as a second arterial graft was performed in 45.4% of our patients. Although the BIMA was strongly correlated with survival in the mono-variate analysis, it lost significance in the multivariable model. This suggested that patients with a higher risk profile might have been less likely to receive BIMAs and that a higher risk profile (older age and PAD) had a more dominant effect in the multivariable model.

The in-hospital mortality was 2.3%, and the 30-day mortality was 2%. A total of 19 (1.71%) strokes occurred in the postoperative period, and 49 (4.41%) patients suffered a postoperative myocardial infarction. Taking into account the high-risk profile of the patients in this cohort, these outcomes could be considered favorable in comparison with those of other published studies [29,30].

As expected, age was a strong independent prognostic factor. Peripheral artery disease was present in 14% of all patients and was an independent predictor of in-hospital mortality after a logistic regression analysis. The increased atherosclerotic burden in these patients may have explained the increased rate of cardiovascular events and poorer outcomes [31].

To the best of our knowledge, this was the largest study to date to evaluate the results of the treatment of CTO patients with CABG. It indicated that CABG remains an excellent treatment strategy with high revascularization rates for CTO and with very favorable clinical postoperative results. Furthermore, this analysis was a strong basis for a comparison with interventional techniques for the same subset of patients.

## 5. Conclusions

This study showed that CABG achieved high revascularization rates and was associated with very favorable in-hospital outcomes in patients with multi-vessel disease and coronary total occlusions. Age and peripheral artery disease were independent negative predictors of in-hospital mortality. A long-term follow-up and the type of graft (arterial vs. venous) used would have provided more useful data on the impact that surgical revascularization had on this group of patients.

## Figures and Tables

**Table 1:** Baseline characteristics.

	All (N = 1111)	Survivors (N = 1086)	Non-Survivors (N = 25)	*p*-Value
Age (years)	68.0 [61.0–74.0]	68.0 [61.0–73.8]	76.0 [70.0–79.0]	<0.001
Male	947 (85.2%)	925 (85.2%)	22 (88.0%)	>0.99
BMI (Kg/m^2^)	27.7 [25.4–30.4]	27.7 [25.4–30.5]	27.7 [24.3–30.0]	0.437
Hypertension	924 (83.2%)	904 (83.2%)	20 (80.0%)	0.595
Diabetes	363 (32.7%)	355 (32.7%)	8 (32.0%)	>0.99
Smoking	431 (38.8%)	422 (38.9%)	9 (36.0%)	0.934
Hypercholesterolemia	903 (81.3%)	885 (81.5%)	18 (72.0%)	0.295
Atrial Fibrillation	91 (8.2%)	87 (8.0%)	4 (16.0%)	0.142
Prior MI	330 (29.7%)	321 (29.6%)	9 (36.0%)	0.634
PAD	155 (14.0%)	146 (13.4%)	9 (36.0%)	0.004
COPD	97 (8.7%)	94 (8.7%)	3 (12.0%)	0.474
Prior Stroke	66 (5.9%)	63 (5.8%)	3 (12.0%)	0.182
No. of Diseased Vessels				>0.99
Two Vessels	58 (5.2%)	57 (5.25%)	1 (4.0%)	
Three Vessels	1053 (94.8%)	1029 (94.8%)	24 (96.0%)	
LVEF < 35%	49 (4.4%)	46 (4.2%)	3 (12.0%)	0.094
CCS Class	N = 840	N = 823	N = 17	0.195
0	82 (9.8%)	80 (9.72%)	2 (11.8%)	
1	41 (4.9%)	40 (4.86%)	1 (5.88%)	
2	270 (32.1%)	268 (32.6%)	2 (11.8%)	
3	319 (38.0%)	312 (37.9%)	7 (41.2%)	
4	128 (15.2%)	123 (14.9%)	5 (29.4%)	
NYHA	N = 840	N = 823	N = 17	0.258
I	65 (7.7%)	65 (7.9%)	(0.0%)	
II	362 (43.1%)	357 (43.4%)	5 (29.4%)	
III	384 (45.7%)	373 (45.3%)	11 (64.7%)	
IV	29 (3.5%)	28 (3.4%)	1 (5.9%)	
	N = 1111	N = 1086	N = 25	
GFR (mL/min)	78.8 [63.0–90.5]	78.8 [62.9–90.6]	79.7 [69.4–87.8]	0.987
Creatinine (mg/dL)	1.0 [0.8–1.1]	1.0 [0.8–1.1]	1.0 [0.8–1.0]	0.949
Urea (mg/dL)	6.0 [5.1–7.2]	6.1 [5.1–7.2]	5.5 [4.6–6.2]	0.056
CRP (mg/dL)	0.3 [0.2–0.8]	0.3 [0.2–0.8]	0.3 [0.2–0.6]	0.900
Leukocytes (1/μL)	7280 [6050;8950]	7280 [6062;8968]	6780 [5890;8110]	0.314
Hemoglobin (g/dL)	14.4 [13.3–15.3]	14.4 [13.3–15.3]	14.2 [13.0–15.2]	0.534
EuroSCORE II	1.43 [1.02;2.33]	1.42 [1.02;2.30]	3.33 [1.56;4.54]	0.001

Table 1. Data are presented as median (q1, q3) for continuous variables and as n (%) for categorical variables. BMI, body mass index; CCS, Canadian Cardiovascular Society; COPD, chronic obstructive pulmonary disease; LVEF, left ventricular ejection fraction; MI, myocardial infarction; NYHA, New York Heart Association; PAD, peripheral artery disease.

**Table 2:** Lesion characteristics and revascularization rates.

	All (N = 1111)	Survivors (N = 1086)	Non-Survivors (N = 25)	*p*-Value
No. of CTOs (mean ± SD)	1.24 (0.45)	1.24 (0.45)	1.24 (0.44)	0.989
No. of CTOs Bypassed (mean ± SD)	1.08 (0.54)	1.08 (0.54)	1.08 (0.57)	0.974
CTO Localization/Distribution *				
RCA	760 (68.4%)	741 (68.2%)	19 (76.0%)	0.543
LCA	1 (0.1%)	1 (0.1%)	0 (0.0%)	>0.99
LAD	293 (26.4%)	288 (26.5%)	5 (20.0%)	0.616
LCX	317 (28.5%)	311 (28.6%)	6 (24.0%)	0.777
RCA-LAD	94 (8.5%)	90 (8.3%)	4 (16.0%)	0.154
RCA-LCX	115 (10.4%)	113 (10.4%)	2 (8.0%)	>0.99
LCX-LAD	30 (2.7%)	30 (2.8%)	0 (0.0%)	>0.99
RCA-LAD-LCX	11 (1.0%)	11 (1.0%)	0 (0.0%)	>0.99
Multiple CTOs	251 (22.6%)	245 (22.6%)	6 (24.0%)	0.098
Bypass to RCA-CTO	677/760 (89.1%)	660/741 (89.1%)	17/19 (89.5%)	>0.99
Bypass to LAD-CTO	273/293 (93.2%)	268/288 (93.1%)	5/5 (100%)	>0.99
Bypass to LCA-CTO	1/1 (100%)	1/1 (100%)	0	
Bypass to LCX-CTO	265/317 (83.6%)	260/311 (83.6%)	5/6 (83.3%)	>0.99
Full CTO Revascularization	957 (86.1%)	935 (86.1%)	22 (88.0%)	>0.99
BIMA	504 (45.4%)	500 (46.0%)	4 (16%)	0.005
Distal Anastomosis (mean ± SD)	3.73 (0.9)	3.73 (0.9)	3.80 (1.1)	0.758
	N = 989	N = 968	N = 21	
CPB Time (min)	88 [75–104]	88 [75–104]	90 [73–128]	0.276
Cross-clamp Time (min)	64 [54–76]	64 [54–76]	62 [55–99]	0.695
Operative Time (min)	210 [190–240]	210 [190–240]	210 [180–250]	0.958

Table 2. Data are presented as median (q1, q3) for continuous variables and as n (%) for categorical variables unless specified otherwise. CTO, chronic total occlusion; LAD, left anterior descendent; LCA, left coronary artery: LCX, left circumflex artery; RCA, right coronary artery; BIMA, bilateral internal mammary artery; CPB, cardiopulmonary bypass; * RCA-/LAD-/LCX-including branches.

**Table 4:** Predictors of in-hospital mortality.

Covariates	Odds Ratio (95% Confidence Interval)	*p*-Value
Age	3.44 [1.81–6.53]	<0.001
Gender	0.60 [0.17–2.10]	0.429
PAD	3.32 [1.39–7.93]	0.007
COPD	0.99 [0.28–3.53]	0.987
Reduced LV Function	0.77 [0.33–1.77]	0.537
BIMA	0.48 [0.15–1.53]	0.218

Table 4. BIMA, bilateral internal mammary artery; COPD, chronic obstructive pulmonary disease; PAD, peripheral artery disease.

## Data Availability

The data underlying this article cannot be shared publicly due to the requirements of our institutional review board. Individual reasonable requests will be evaluated by the corresponding author.

## Abbreviations

CAD—coronary artery disease; CABG—coronary artery bypass surgery; CTO—chronic total occlusions; BIMA—bilateral internal mammary artery.
